# Listening comprehension in a home language: a case of Russian in Germany

**DOI:** 10.3389/fpsyg.2024.1426831

**Published:** 2024-10-22

**Authors:** Luca Gacs, Anna Ritter, Evghenia Goltsev

**Affiliations:** Department of Arts and Humanities, Institute of German Studies, University of Koblenz, Koblenz, Germany

**Keywords:** listening comprehension, levels of comprehension, home language maintenance, bilingual family, second migrant generation

## Abstract

Listening comprehension is central to language learning, yet it remains the least understood and least researched skill. This statement is still relevant today, as there is insufficient research to explore listening comprehension from the perspective of family-related multilingualism and to consider the complete linguistic repertoire of multilingual speakers. Moreover, with regard to home language, listening comprehension is assumed to be a more developed language competence than reading or writing. Based on the mentioned research, the aim of the present study is to investigate listening comprehension and its influencing factors specifically in German-Russian simultaneous bilinguals aged 13–19 (*n* = 99) by considering the home- and majority language. The study uses quantitative data collection methods such as linguistic tests in Russian and German for the elicitation in different levels of listening and questionnaires for strategy use and background. The research questions are as follows: What does the language proficiency and input in Russian look like? (1) Regarding listening comprehension in Russian as a home language, are there differences between the speakers within comprehension on different levels, e.g., is sound decoding easier than sentence parsing? (2) If there are differences in Russian as a home language, which linguistic and background variables can correlate with the performance of listening comprehension on its different levels? Concerning the first research question, the results show relevant differences between four different levels of listening comprehension (phoneme, word, sentence, and text level), which strengthened the assumed complexity of listening comprehension in the home language. In addition, the results show different connections between the listening comprehension competence and the input from different family members, as well as exposure to film and television in the home and majority language.

## 1 Introduction

Hearing is the first sense that a child develops after birth and, thus, is decisive for the phonetic development of the first year of life (Eckhardt, 2020). In addition to oral communication, listening comprehension provides access to a vast variety of video and audio data via modern media (starting from radio and TV to social networks, podcasts, blogs, and YouTube) (*cf.* Vandergrift, 2007). Given that, listening comprehension is crucial to language learning regardless of whether it is a first (L1), second (L2), or foreign language (Grosjean and Byers-Heinlein, 2018). In the school context, listening means the reception of instructional and media input on the one hand and direct communication with other speakers on the other, as teaching is strongly communicative and orally oriented (Becker-Mrotzek and Vogt, 2009). Around two-thirds of lesson time is dedicated to oral input and exchange (Lengyel, 2012; Ahlers et al., 2009).

However, a number of researchers state that while listening is a relevant and important competence for language teaching at school and other education institutions, it still needs more research (e.g., Vandergrift, 2007; Eckhardt, 2020; Dietz, 2017; Vandergrift and Baker, 2015; Namaziandost et al., 2019). Moreover, there is not enough research to explore listening comprehension from the perspective of family-related multilingualism, transfer, and preservation of heritage languages from the sociolinguistic perspective, which considers the complete repertoire (e.g., Gervain and Werker, 2013; Byers-Heinlein, 2018; Adamczak-Krysztofowicz and Limbach, 2019). Nevertheless, with regard to the home language, listening comprehension is assumed to be a more developed language competence than reading or writing (Mehlhorn and Rutzen, 2020). Still, many questions in this field remain open and more research is necessary, especially considering the entire process of listening, from the decoding of sounds and word recognition to the parsing of whole sentences and understanding of whole texts, and the influencing factors of the entire process of listening comprehension (e.g., Grosjean and Byers-Heinlein, 2018) and its influencing factors in the context of family-related multilingualism.

This is exactly the focal point of the study at hand, as the research described in this article aims to contribute to the knowledge concerning listening comprehension from the perspective of family-related multilingualism. Additionally, it focuses specifically on Russian as a home language in Germany, considers the different levels of listening comprehension that contribute to the understanding of the complex process of listening, and the linguistic repertoire of simultaneous bilingual adolescents. Therefore, the study aims to capture the listening process as a whole in 99 Russian-German bilinguals aged 13–19. This particular age group was selected because the present study may give the basis for further pedagogical studies on bilingual students belonging to the second immigrant generation. The research questions for the present article focus on the listening comprehension in Russian as a home language and are as follows:

RQ1: Regarding listening comprehension in Russian as a home language, are there differences between the speakers within comprehension on different levels, e.g., is sound decoding easier than sentence parsing?

RQ2: If there are differences in Russian as a home language, which linguistic and background variables can correlate with the performance of listening comprehension at different levels?

To answer these research questions, our analysis used several listening tasks on the phoneme, word, sentence, and text level. These tasks were mostly developed based on studies that research listening comprehension focusing on different home languages in Germany (Brehmer and Mehlhorn, 2015; Gogolin et al., 2017; Edele et al., 2012, 2015) as well as on the existing theoretical framework of listening comprehension in the first and foreign language (e.g., Vandergrift, 2007; Field, 2008; Grosjean, 2018). However, the specific set of the listening tasks is unique, was developed in accordance with the research questions of the present study and uses descriptive as well as suitable inferential statistical research methods.

In addition to this Introduction, the article consists of five sections. Section 2 provides a literature overview on the main relevant points of the article and outlines a contextual background of Russian as a home language in Germany. Section 3 follows with the description of the study at hand. Section 4 focuses on the study results with regard to the research questions, while Section 5 discusses and summarizes the main findings, and statistical data, providing future research directions based on the present and other existing studies in the area of inquiry.

## 2 Literature overview and contextual background

### 2.1 Migration and home languages

In recent decades, an increasing number of scientific studies have focused on the area of language development in multilingual families, including multilingual immigrant families (e.g., Schwartz and Verschik, 2013; Lanza, 2021; Juvonen et al., 2020; Mayer et al., 2020; Schwartz, 2020; Karpava, 2022; Wald et al., 2023; Zabrodskaja et al., 2023). In this process, a series of overlapping and interconnected terms emerged describing the language or languages spoken in multilingual families. These are majority and minority languages, societal and home or community language, dominant and heritage language just to name some of them (see more in Schalley and Eisenchlas, 2020). However, all these pairs of terms have in common that communication in the multilingual families is characterized by the use of at least two languages which play different roles. In the present article it was chosen to refer to the term “home language” which is defined as follows. Home languages are “languages spoken or used in the home or community but which are not the majority language in the society” (Connaughton-Crean and Ó’Duibhir, 2017).

The key factor for the acquisition and maintenance of the home language is the family, meaning both the nuclear family and the extended family with grandparents and second-degree relatives, as family plays the crucial role in the process of the socialization (*cf.* Melo-Pfeifer, 2015; Karpava, 2022). In the course of the family language policy (FLP) different strategies and ideologies concerning the process of learning, managing and use of the home language are negotiated between family members.

Hereby, a number of internal and external factors may have an impact on the language development within the family. The internal factors include the parental language attitudes, expectations, and efforts (i.e., parental input), the family constellation (parental languages), the age of arrival of the parents, language management strategies in the family, children agency, the number of children and their age difference (*cf.* Riehl, 2018; Zabrodskaja et al., 2023). To the external factors belong societal regulations of minority languages and their use, schooling and further education, socioeconomic status of the family, speaker community and its social network, quality, and quantity of input on the home and majority language outside the home environment (*cf.* Juvonen et al., 2020; Zabrodskaja et al., 2023).

### 2.2 Russian as a home language in Germany

Modern Germany is a country of immigration where many different languages are spoken at home and/or at work, in addition to German. A number of immigrant communities in Germany have a long history and consist of two or three generations, for example, immigrants from Poland, former Yugoslavia, Italy, Greece, and Turkey. The community of Russian-speaking immigrants is one of the largest and comprises at least two generations. Its history started in the 1950s and received a new boost at the end of the 1980s when thousands of people immigrated to Germany from the Soviet Union and later from its successor states (Dietz and Roll, 2019). Their migration was generally triggered by the profound political transformation processes and the subsequent collapse of the Soviet Union (*ibid.*).

Although the immigrants came from different countries like Russia, Kazakhstan, or Kyrgyzstan, most of them were raised in the soviet culture with more or less the same state language policy (Grenoble, 2003; Brüggemann, 2019) and therefore, considered Russian to be their first or one of the first languages. At the same time, the great majority of Russian-speaking immigrants had little or no knowledge of German by the time of their arrival (Baur et al., 2019). Over the course of more than three decades, an extensive and widespread Russian-language infrastructure has been built up in Germany, including private and partly state-founded Russian language education (*cf.* Ritter, 2021). On the one hand, this infrastructure is used by the migrants and, on the other, many work in it and are part of it, which contributes significantly to the maintenance of the Russian language. It can therefore be assumed that the second and, in some cases, the growing third generation of Russian-speaking migrants come into contact with the Russian language not only exclusively within the family but also in public, especially orally, through listening. Furthermore, they may have a possibility to learn Russian as a foreign language at school or in private institutions, which strongly depend on the educational policy of a federal state (Zabrodskaja et al., 2024). Despite the wide use of the Russian-speaking infrastructure, the context, frequency and language proficiency of Russian in immigrant families is rather heterogeneous and depends on factors like the migrant history and the language attitudes of family members.

### 2.3 Listening and multilingualism

The first findings about bilingual listening comprehension and multilingualism have their source in the second half of the 20th century when techniques were developed to measure bilingual listening comprehension abilities as an indicator for the degree of bilingualism (Cooper et al., 1969). Still, the main focus of the listening comprehension research was on the process during foreign language acquisition and less on the first language listening process, primarily because proficient listeners often conduct the listening processes automatically, so that the mechanisms and efforts stay hidden (Adamczak-Krysztofowicz and Limbach, 2019; Vandergrift, 2007; Dietz, 2017). Moreover, listening comprehension has been regarded as self-developing for the longest time and research concerning this topic was therefore not considered to be of primary importance (Belgrad et al., 2008). However, listening plays an important role within foreign language didactics and research, especially since the communicative turn in the 1970s that introduced the approach of communication-oriented language teaching (*cf.* Suemith, 2011). In addition, the research on listening comprehension is part of psycholinguistic research (e.g., Valentini and Serratrice, 2023). Nevertheless, especially from a sociolinguistic perspective, a number of questions connected to listening comprehension remain open, among others the differences between the speakers within comprehension on different listening levels and the influencing factors of listening comprehension in specific language combinations.

Notwithstanding that, listening is an active process that helps to form meaning from auditory input and to link it to prior knowledge in order to create mental representations of what has been heard (Namaziandost et al., 2019). Listening comprehension is very complex and multidimensional and consists of several psychological and cognitive processes. It is performed in real time which is particularly difficult because the working memory has limited capacity and information that is not processed immediately cannot be revised (Dietz, 2017; Field, 2008; Imhof, 2010; Vandergrift, 2011).

Among others, Field (2008) differentiates between the product and the process of listening comprehension. By the term “product” the final stage of listening comprehension is meant, i.e., understanding the whole text. Every single step that leads to the final comprehension of a text or other auditory input can be referred to as the process of listening comprehension (Field, 2008; Vandergrift, 2011). The listening process consists of different components that contribute to the final stage of understanding the whole spoken input. In addition to linguistic knowledge and other sources of knowledge, various processing mechanisms are used until the mental representation of what is heard can be created. This works incrementally (Grosjean and Byers-Heinlein, 2018).

On the one hand, processing mechanisms include the segmentation of the sound stream and decoding it, beginning from the smallest units (Imhof, 2010). In the beginning, the first sounds that are heard are categorized, and the phonemes and syllables of that language that is heard are identified. After that word recognition begins. In this step several words are activated in the mental lexicon that fit to the sounds heard and compete with each other. The candidates are evaluated repeatedly and are narrowed down based on the proceeding input until the selection of one word is possible (Cutler, 2012; Cutler and Broersma, 2005; Dietz, 2017). The identification of words then opens the access to information stored in the mental lexicon that helps with syntactic parsing and allows to decode the sound stream further until sentences, larger speech units, or even texts can be understood. These mechanisms are called bottom-up listening processes (Adamczak-Krysztofowicz and Limbach, 2019).

On the other hand, meaning is also constructed by using other cognitive processes and prior world and situational knowledge. These mechanisms are called top-down processing mechanisms and help to fill gaps that are left by bottom-up decoding processes. Therefore, the two main types of processing mechanisms complement each other (Field, 2008; Vandergrift, 2011). However, which process is prioritized in processing depends on many situational and individual factors of the listener’s situation, such as the listening intention or the context in which the listening intention or the context in which the listener finds him/herself (Vandergrift, 2007). In any case, the processing of speech input is predictive (Grosjean and Byers-Heinlein, 2018). Although the mechanisms of listening comprehension have been researched over time, it is still the least understood of all language skills and many questions remain open (Namaziandost et al., 2019). Among others, the question of how much the different levels of processing influence each other is highly debated. While some researchers suggest a highly interactive approach where all levels of processing influence each other from the very start, others assume that at least some of the processes operate independently of each other (Grosjean and Byers-Heinlein, 2018).

There are also many factors that influence its outcome, e.g., different types of knowledge, such as background knowledge and topic familiarity (Othman and Vanathas, 2017), as well as linguistic knowledge. For foreign language acquisition, not only the vocabulary size in the L2 is important but also the vocabulary size in the L1 and listening comprehension in the L1 can have an impact on the final product of listening comprehension (Vandergrift, 2006; Vandergrift and Baker, 2015). Additionally, the morphosyntactic competence seems to have an impact on listening comprehension (Marx and Roick, 2012). Listening comprehension is a cognitive process, which means that other skills have an impact on its outcome, such as metacognition, behavioral attention, the working memory, strategy usage, and the ability of auditory discrimination (Namaziandost et al., 2019; Jiang and Farquharson, 2018; Vandergrift and Baker, 2015; Wilson et al., 2011). However, language proficiency seems to have the biggest impact on the product of listening comprehension (Vandergrift, 2006, 2007). This effect is also stated for heritage speakers in their L2 (Marx and Roick, 2012).

In the context of multilingualism, most of the research originates in the field of second or foreign language acquisition (Dietz, 2017; Namaziandost et al., 2019). Other studies consider only one language, namely the majority language. This is especially the case in large-scale studies, for example, national educational panels, such as the IQB Trends in Student Achievement (Stanat et al., 2022). Even more, in most of the studies only the final result of listening comprehension is considered without paying attention to the process (Edele, 2016). In contrast, only a few studies deal with listening comprehension in the home language (Anstatt and Mikić, 2022; Brehmer and Mehlhorn, 2015; Marx and Roick, 2012; Mehlhorn and Rutzen, 2020).

Listening comprehension has the same structure in multilingual as in monolingual first language listening. It consists of the same sub-processes and uses top-down and bottom-up processing mechanisms (Marx, 2016). However, there are differences in the way these processes are implemented at the various levels of processing of auditory input since there are cognitive differences between people who are exposed to two or more languages and people who are raised in a monolingual environment across the lifespan (Barac et al., 2016; Bialystok and Craik, 2022). These differences may be linked to the fact that the languages of multilinguals cannot be considered as separate systems but are always influencing each other (Riehl, 2015; Van Dijk et al., 2022). Therefore, the components of listening comprehension in multilinguals should be multiplied by the number of languages they speak, and one or more languages have to be deactivated in order to be able to process monolingual speech in one language (Grosjean and Byers-Heinlein, 2018).

These differences have an impact on the factors influencing the different levels and processing mechanisms of listening comprehension, such as different types of knowledge (linguistic and world knowledge), skills, and strategies. Hence, among the influencing factors that are crucial to listening comprehension in monolinguals, many more aspects come into play, especially when the home language is different from the majority language. In this case the family environment and language use may also be important influencing factors, since there is a significant impact on listening comprehension concerning the language biography of the multilinguals, the usage and purpose of their languages, and language proficiency (Gervain and Werker, 2013; Byers-Heinlein et al., 2017; Gorsjean and Byers-Heinlein, 2018). However, language proficiency (mostly vocabulary size, but also grammar) is stated to have the biggest effect on successful outcomes (Marx and Roick, 2012; Vandergrift, 2006, 2007; Vandergrift and Baker, 2015). This is the reason why, in addition to the impact of background factors (e.g., amount of input, amount of active usage of the language, education of parents, media consumption), the study at hand focuses on the link between language proficiency and listening comprehension in Russian, being the home language of the participants.

## 3 Study and methodology

### 3.1 Participants and design

The present study was organized at the University of Regensburg and the University of Koblenz and conducted in different states and cities in Germany, mainly in Bavaria during the year 2023. It comprised two sets of tests and a background questionnaire for the main group of participants: in Russian and in German.

The study at hand was focused on Russian-German bilingual adolescents between 13 and 19. The mean age was 15.92. Due to the analysis procedures, our goal was to conduct the tests with 99 participants. A certain high level of Russian was not a requirement for the participation in the study.

However, we tried to make sure by a background questionnaire and certain tasks on Russian language proficiency, that the participants encountered Russian input on a daily basis as a home language. Thus, the self-assessment was only an additional variable. Since the study focuses on oral skills, the command of the Cyrillic alphabet was not required. Most of the participants were born in Germany into Russian-speaking immigrant families, some immigrated to Germany at the age of three or younger. Thus, all of them belong to the second or third immigrant generation. All participants attend public German schools where German is the language of education.

The tests were generally carried out on two dates. Listening comprehension in German was tested on 1 day and Russian listening comprehension on the other. The testing was conducted individually. The tasks and the procedure were identical in both languages. Namely we began with assessing the language proficiency (1; Table 1) in the respective language after that the testing of the products of four different levels of listening comprehension (3–6) took place, and finally, the strategies (7) were assessed (though these results are not included in the present article). Each participant was provided with a laptop and a pair of headphones. The questions, i.e., listening tasks (except the listening task on the text level), were embedded into a PowerPoint presentation and the answers had to be filled in on a separate sheet of paper.

**Table 1:** Overview of the instruments used in the study.

1	Background data	Questionnaire (MEZ)
2	Language proficiency	Grammar: cloze test (Brehmer and Mehlhorn, 2015 and MEZ) Vocabulary: picture test (Brehmer and Mehlhorn, 2015)
3	Product of listening comprehension on phoneme level	Short listening exercises (own development)
4	Product of listening comprehension on word level	Short listening exercises (own development)
5	Product of listening comprehension on sentence level	Short listening exercises (own development)
6	Product of listening comprehension on text level	Listening exercises (from LlfBi; NEPS)
7	Strategies on phoneme, word, sentence, and text level	Questionnaire based on Chen (2010) and Nix (2016)

### 3.2 Instrument

Table 1 below gives an overview of the instruments used in the study.

In order to capture influencing factors and confounding variables, a background questionnaire was created. This questionnaire covers questions about aspects such as age or gender, country of birth or the parents’ education, and attitudes toward the Russian language. There are also questions about the language background of the participants. Such as which languages are spoken at home and which language they speak with different family members or peers, but also which languages are spoken with them by their parents. In addition, questions about their use of different medial input in German or Russian are to be found in the background questionnaire. Most of the elements are taken from the Multilingual Development: A Longitudinal Perspective (MEZ for Mehrsprachigkeit im Zeitverlauf) project,^1^ some additional questions were included as well. The questionnaire was sent to the participants in advance together with all the information to the study.

The two tasks used for assessing the language proficiency in Russian and German were already used in two different projects. The Russian cloze tests were provided by the MEZ project,^2^ the German cloze tests^3^ as well as the vocabulary tests were previously used in the project “Russian and Polish Heritage Language as Resource in the Classroom” (Brehmer and Mehlhorn, 2015). The vocabulary task contained 20 pictures that had to be named in German or Russian depending on which language the test was conducted in. The cloze tests for each language were used to test the grammar skills.

As for testing the product, i.e., results of the listening comprehension on the different levels, different tasks were used. The listening tasks for the text level were from the National Educational Panel Study (NEPS) and are owned by the Leibnitz Institute for Educational Trajectories (LlfBi) and last 30 min for each language. The German version consists of different listening tasks, the test items are multiple choice items and are presented twice, and the listening texts cover authentic spoken and written language (Hecker et al., 2015). The texts in the Russian version were originally created in German and then translated to Russian and cover a broad range of language proficiency and text types. The test items consist of multiple-choice items. Prior to the Russian listening comprehension test, the participants worked on a task that contained eight easy sentences that had to be linked to one out of five pictures (Edele et al., 2012, 2015). Both the Russian and German listening tasks were adapted without any changes, as they were established for and tested in large-scale studies of the National Educational Panel.

Additionally, different tasks were developed within the study and by the research team on the phoneme and syllable, word, and sentence level and were embedded into a PowerPoint presentation that the participants could navigate individually on their laptops. The tasks mostly consisted of an audio recording and the identification of the corresponding picture(s) or word(s). The audio examples for the majority of the tasks were recorded in an audio laboratory by authentic native speakers of Russian and German. There were four to five tasks on every level for each language testing different peculiarities of both languages.

On the phoneme level, it was aimed to find phonetic contrasts that result in different lexical meanings in one language but not in the other. Therefore, there were tasks that tested phoneme decoding, such as the identification of long and short vowels in German or the discrimination of palatalized or non-palatalized consonants in Russian. Furthermore, there was a task on the pronunciation of the voiceless uvular and velar fricative, as well as the voiceless fricative in German, as these sounds do not exist or are not differentiated in Russian and one task on vowel reduction as a distinctive phenomenon in Russian, but not in German. Additionally, there was a task each on recognition of acceptable syllables and rhyme words in both languages and one syllable separation task.

On the word level, there was one listening task for the identification of homographs with different pronunciation, one task for the identification of homophones and one task on acceptable and non-acceptable prefixes both in German and Russian. In addition, there was a task in both on grammatical gender, as this is contrasting in both languages.

The sentence level covered tasks of emotion and intonation recognition, and the identification of different sentence types, since they vary in intonation in Russian and German. In addition, the identification of syntactic functions and stressed words within a sentence were tested by picture assignment tasks.

### 3.3 Statistical methods used

The evaluation of results is mainly quantitative with descriptive statistical means and further statistical tests such as repeated measures ANOVA and linear regression models. The tests were carried out in the statistics program IBM SPSS. For the analysis, the significance level of *α* = 0.05 is set as a minimum requirement.

The normal distribution of the data is assumed due to the size of the sample following the central limit theorem (*cf.* Islam, 2018). For the numeric coding of the data for each correct answer one point was given at the various levels of listening comprehension. In the calculations, either the total scores or, for better comparability, the percentage values of the total scores were used. Most of the background variables were coded using Likert scales with one as the lowest and four or five as the highest value. The questions regarding the language use with different contact persons are coded from “only German,” “mostly German,” “both languages equally” to “mostly Russian,” and “only Russian.” The questions concerning the media use in both languages are coded on a five-point Likert scale from “not true at all” to “exactly true.” The questions in context of language attitudes are coded on a four-point scale using the same labels.

The educational background of the parents is divided into two nominal categories: “completed professional training without university degree” and “completed university degree.” As for the variables that measure language proficiency, the results of the vocabulary test and the cloze tests are taken. Every correct answer equals one point, these are summed up and build the final scores for the variables vocabulary and grammar skills.

For research question one, the percentage values of the scores achieved on the different levels of listening comprehension were used. After a short overview using descriptive statistics, a repeated measures ANOVA is calculated (*cf.* Park et al., 2009), since it is assumed that the different levels of listening comprehension correlate with each other and cannot be viewed as independent variables. Mauchly’s test will be used to ensure that sphericity is met. If this is not the case, the degrees of freedom will be corrected using Greenhouse–Geisser or Huynh-Feldt. To examine the differences between the levels in detail, a post-hoc pairwise comparison with a Bonferroni adjustment is used. In addition, a multiple linear regression model (*cf.* Eberly, 2007) is built in order to check which of the sub-levels of listening comprehension has the strongest influence on the text level. The absolute value of the final results of the text level listening comprehension is used as the dependent variable. The absolute values of the scores achieved on the phoneme, word, and sentence level are used as predictor variables.

For the research questions, two Pearson correlations are conducted for measuring the relation between the background variables, the linguistic input, and the results on the different levels of listening comprehension. For these calculations, the absolute scores achieved on the phoneme, word, sentence, and text level are taken as indicators of the listening comprehension on the respective levels. In the case of the correlations with the educational background of the parents, Spearman correlations are used instead of Pearson, because the scale of this variable is nominal (*cf.* Diekmann, 2017). The correlation coefficients are interpreted according to Cohen (1988)’s guidelines.

The following variables are considered in the analysis of relations between the results of the listening comprehension, linguistic, and background variables:

- Parents’ education
- Input: the amount of Russian spoken by the parents to each other, by the mother and father spoken to the participant, total amount of passive input in Russian (mean value of the three variables).
- Amount of Russian spoken by the participant with the mother, father, grandmother, grandfather, siblings, friends, fellow students, and acquaintances as well as the total amount of Russian spoken with these contact persons (mean value of these values).
- Attitude toward Russian: the mean value of the importance of the Russian language to the participant and the importance of language proficiency in Russian for the participant.
- Media consumption in Russian and German: amount of input from television and films, music, and social media in Russian and German as well as the total amount of Russian media consumption (mean value of the three variables).

## 4 Results

### 4.1 Background variables and language proficiency

A total of 99 valid data sets were included in the study. The participants stated that they had acquired oral knowledge of Russian mostly through parental input. One third (*N* = 34) of the participants take Russian classes at school. However, most of them had no command of the Cyrillic alphabet at all.

The participants were asked to evaluate their knowledge of Russian and German and the frequency of their communication in Russian, i.e., with their family members, friends, and in the media.

The questions relating to linguistic knowledge were divided into four categories. The participants assessed their skills in the areas of “understanding,” “speaking,” “reading” and “writing” in both languages on a scale from one to six. The scale is based on the German grading system, in which 1 is the best and 6 the worst grade. The question concerning “understanding” was assessed by 96 participants in Russian and 97 in German. “Speaking” was evaluated by 95 participants in both German and Russian. A total of 95 participants rated their reading skills in Russian and 96 in German. The ability to write was assessed by 94 participants in Russian and by 95 participants in German. Table 2 shows the self-evaluation of German, Table 3 the evaluation of Russian skills.

**Table 2:** Self-assessment of German skills in percent.

Grade	Understanding	Speaking	Reading	Writing
1	79.4	55.8	64.6	45.3
2	15.5	36.8	24.0	27.4
3	3.1	5.3	9.4	16.8
4	0	0	0	8.4
5	0	0	0	1.1
6	2.1	2.1	2.1	1.1
Mean	1.32	1.58	1.53	1.96
*N*	97	95	96	95

**Table 3:** Self-assessment of Russian skills in percent.

Grade	Understanding	Speaking	Reading	Writing
1	34.4	21.1	16.8	3.2
2	42.7	34.7	19.8	12.8
3	16.7	29.5	20.0	14.9
4	3.1	12.6	21.1	25.5
5	3.1	2.1	13.7	20.2
6	0	0	11.6	23.4
Mean	1.98	2.40	3.33	4.17
*N*	96	95	95	94

Overall, they stated to be more advanced in the German language than in Russian. In Russian, the participants stated to have primarily oral competences rather than written ones. In German, the receptive language skills were ranked higher than the productive skills. However, self-assessments were not included into the further analysis, as these variables for language proficiency are not reliable enough.

On average the participants scored 15.30 out of a total of 20 points in the vocabulary test (Table 4). The standard deviation was approximately 5.01 points. As for the grammar skills, the participants reached 40.23 out of a total of 56 points on average. The standard deviation lies around 13.10.

**Table 4:** Russian language competence.

	*N*	Minimum	Maximum	Mean	Standard deviation
Vocabulary	99	0	20	15,30	5,098
Grammar	99	0	56	40,23	13,099

With regard to language attitudes, the participants were asked whether Russian is important to them. 57.6% of the participants fully agreed and 31.3% partially agreed. Regarding whether a high level of proficiency in Russian is important to them, full agreement was stated again in 57.6% of the cases and partial agreement was indicated in 31.1% of the cases. Only 1.0% answered with “not true at all” in both cases. For further calculations, the mean of the responses to both questions was built.

The participants were asked about their use of German and Russian media. Only 6.1% of all participants do not watch any films or television in German, whereas Russian television and films are not consumed in 15.2% of the cases. The participants fully agreed to the question whether they listen to Russian music in 38.4% of the cases and they agreed to the question of listening to German music in 24.2% of the cases. No Russian music is listened to by 16.2% of the participants and 15.2% of the participants stated to never listen to German music. No German social media is consumed in 5.1% of the cases and no Russian social media is consumed by 20.2% of the participants. 16.4% of the participants stated that they do not speak any German at home at all. 29.3% indicated to use more German and 51.5% indicated to use more Russian daily at home, the rest did not answer the question.

Table 5 shows the exact percentage of the amount of German and Russian used by the parents in communication with each other, and the language used by the mothers and fathers addressing the participants (participants are referred to as PT). In sum, over three quarters of the participants stated that their parents talk only Russian or mostly Russian to each other. In about 80% of the cases, the mother talks only Russian or mostly Russian with the participant. Fathers tend to give a little less input in Russian, but a little more input in German than mothers. In 69.7% of the cases, the father talks only Russian or mostly Russian with the participant. While mothers use only or mostly German in 8.1% of the cases, fathers use the German language (mostly or only) in 20.2% of the cases with regard to the communication with the participants.\.

**Table 5:** Amount of input in German and Russian given by the parents in percent.

Language spoken	Parents to each other	Mother to PT	Father to PT
Only German	6.1	1.0	11.1
Mostly German	4.1	7.1	9.1
Both languages	5.1	9.1	4.0
Mostly Russian	20.2	36.4	23.2
Only Russian	61.6	43.4	46.5
No answer	3.0	3.0	6.1

Communication within the family is mostly in Russian, as 56.6% of the participants talk only Russian or mostly Russian with their mothers and fathers. In 62.6% of the cases communication with the grandmothers is done in Russian and in 52.5% of the cases the participants stated to communicate only in Russian with their grandfathers. In the extended family, the participants tend to communicate only in German or mostly in German with their siblings only (55.5%).

In general, these results of Tables 5, 6 show that the Russian language is highly present in the participants’ everyday life as they get regular input from both fathers and mothers and speak Russian with other family members. These were counted as relevant factors for the present study. Furthermore, this information was used for the statistical analysis of correlating factors with the results of listening comprehension on different levels.

**Table 6:** Language spoken by the participant to family members.

Language spoken by PT to	Mother	Father	Grandmother	Grandfather	Siblings
Only German	6.1	14.1	5.1	5.1	33.3
Mostly German	20.2	18.2	4.0	4.0	22.2
Both languages	13.1	4.0	2.0	3.0	8.1
Mostly Russian	30.3	25.3	13.1	11.1	8.1
Only Russian	26.3	31.3	62.6	52.5	6.1
No answer	4.0	7.1	13.1	24.2	22.2

Apart from German and Russian, other languages are also spoken in the families, like Ukrainian or Kazakh.

Outside of the extended family, the use of German is predominant. It is notable that the participants almost do not use Russian at all at school with fellow students. They tend to use mostly Russian with their friends in 2.0% of the cases. In communication with acquaintances, some Russian is used, but with 56.6% the use of only and mostly German outweighs (Table 7).

**Table 7:** Language spoken by the participant outside of the family.

Language spoken by PT to	Friends	Fellow students	Acquaintances
Only German	45.5	73.7	28.3
Mostly German	26.3	15.2	28.3
Both languages	17.2	7.1	16.2
Mostly Russian	2.0	0.0	16.2
Only Russian	0.0	0.0	6.1
No answer	9.1	4.0	5.1

The use of languages seems to be split into the extended family, where Russian is the main language of communication in most of the cases, and outside of the family, where German seems to be the preferred language of communication. These results are partly in line with previous studies on Russian as a home language in Germany (e.g., Brehmer and Mehlhorn, 2015; Ritter, 2021; Wald et al., 2023; Zabrodskaja et al., 2023). Besides, the participants show remarkably high results in the vocabulary and grammar tests for Russian language as well as a relatively high percentage concerning the use of Russian-speaking media which may have a positive impact on their listening comprehension in Russian.

Apart from the questions concerning the participants themselves, a few questions were asked about the parents and their sociolinguistic background, e.g., their degree of education (*cf.* Schwartz, 2020). Thus, two categories were created for the parents’ educational background. The parents either “completed professional training without a university degree” or “a completed university degree.” In the 56 cases that fit in one of these categories, 42.9% completed professional training without a university degree and 57.1% attained a university degree in Germany.

### 4.2 Research question 1: listening comprehension on different levels in comparison

The first research question dealt with differences between the participants regarding the comprehension on different levels, i.e., phoneme, word, sentence, and text level. It was examined whether sound decoding for example is easier than sentence parsing.

**Figure 1:**
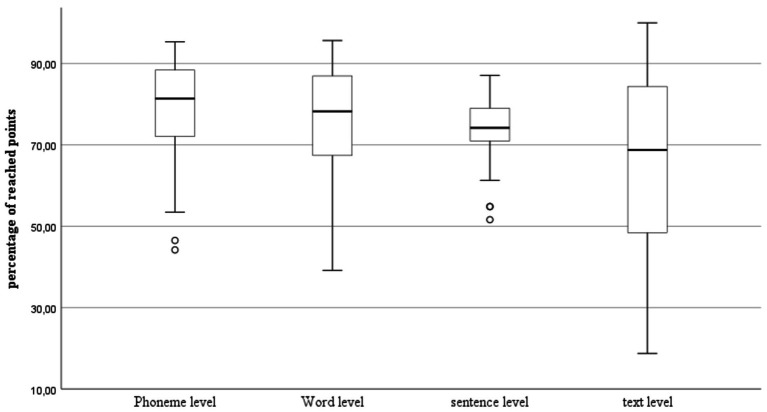
Scores reached by the participants on the different listening tasks in percent.

As shown in Figure 1 below, the participants scored highest on the phoneme level of listening comprehension with an average of 79.57% of the correct answers. The standard deviation was approximately 11.41. The word and sentence level appear to be similar, since the participants reached 75.71% on the word level and 74.78% on the sentence level of listening comprehension. However, the standard deviation was higher on the word level at 12.12 and much lower on the sentence level at 6.76. The participants scored the lowest on the text level of listening comprehension with an average of 66.67%. The variability on this level of comprehension was the highest among the participants. The standard deviation is around 21.18.

A repeated-measures ANOVA was performed to evaluate the difference in the percentage of correct answers in the listening comprehension tests on the different levels. Maulchy’s test indicated that the criterion of sphericity has been violated, 2(5) = 69.37, *p* ≤ 0.001. Thus, the degrees of freedom were corrected using Greenhouse–Geisser estimates of sphericity (= 0.67). The differences in mean percentage of scores were significant at the 0.05 level, *F*_(2, 55.28)_ = 5.70, *p* ≤ 0.001, partial *η*^2^ = 0.164.

Post-hoc pairwise comparison with a Bonferroni adjustment indicated that the percentage of correct answers was significantly higher on the phoneme level than on word level (*p* = 0.009) and sentence level (*p* = 0.001). There was no significant difference in percentages reached at word level and sentence level (*p* = 1.00) though. The scores were lowest on the text level of listening comprehension. They were significantly lower than the results on the phoneme level, word level and sentence level (*p* = 0.001).

Regarding the influence of the phoneme, word, and sentence level on the comprehension of the complete text a multiple linear regression analysis was conducted. Multicollinearity diagnostics were performed to ensure the independent variables were not highly correlated. The regression model delivers a significant regression [*F*_(3, 95)_ = 17.76, *p* ≤ 0.001]. The *R*^2^ was 0.36, indicating that the scores on phoneme, word and sentence level explained approximately 36% of variance in the scores measuring the comprehension of the whole text. The results are shown in Table 8 below.

**Table 8:** Linear regression model for the influence of the different levels on the comprehension of the whole text (within the intercept).

	Dependent variable: text level							Collinearity statistics	
	*b*	SE	*β*	*t*	*p*	95% CI			
						LL	UL	Tolerance	VIF
Intercept	−12.370	6.335		−1.953	0.054	−24.947	0.207		
Phoneme level	0.396	0.136	0.286	2.902	0.005	0.125	0.666	0.693	1.443
Word level	0.889	0.239	0.366	3.714	0.000	0.414	1.365	0.695	1.439
Sentence level	0.202	0.307	0.062	0.656	0.513	−0.408	0.812	0.747	1.339

Significant results were found for the influence of scores on the phoneme level (*p* = 0.005) and the word level (*p* < 0.001). The linear regression model did not deliver significant results for the influence of the scores on sentence level on the scores on the text level of listening comprehension. For each one point on the phoneme level, the predicted scores on the text level of listening comprehension increased by approximately 0.40 points and every increase of score on the word level by one-point results in an increase of score by approximately 0.89 points on the text level of listening comprehension. Therefore, a weak positive influence of word and phoneme level scores on the comprehension of the whole text could be attested with = 0.29 (phoneme level) and = 0.37 (word level).

### 4.3 Research question 2: relevant linguistic and background variables on the different levels of listening comprehension

The second research question aimed to find correlations between different background variables and the results of listening comprehension on the different levels. The link between language proficiency (results of the vocabulary and grammar test) and the results of listening comprehension was also examined. The results below show only significant correlations on each level of listening comprehension. Values with *r* < 0.3 are excluded since they represent weak to negligible correlations (*cf.* Cohen, 1988).

#### 4.3.1 Significant results on the phoneme level

The results on the phoneme level of listening comprehension show a highly significant strong correlation with both of the indicators for language proficiency. The correlation coefficient between the phoneme level and the results on the vocabulary test is *r* = 0.532 and between the phoneme level results and the results of the cloze tests *r* = 0.622. Significant but weak correlations could be found between the results on the phoneme level, the Russian input given by the mothers, and between the amount of Russian the participants talk to their grandfathers and siblings. All the results have a significance level of *p* < 0.01. The results are shown in Table 9.

**Table 9:** Correlations with linguistic and background variables on the phoneme level.

	Phoneme level	Vocabulary	Grammar	Mother to PT	PT to grandfather
Vocabulary	0.532^**^				
Grammar	0.622^**^	0.673^**^			
Mother to PT	0.389^**^	0.508^**^	0.611^**^		
PT to grandfather	0.360^**^	0.473^**^	0.646^**^	0.563^**^	
PT to siblings	0.420^**^	0.404^**^	0.555^**^	0.455^**^	0.322^*^

** < 0.01; * < 0.05.

No significant correlations could be attested for the language used by the fathers and concerning the participants’ communication language with the grandmothers, although the fathers and grandmothers belong to the nearest relatives.

Concerning the education of the parents, a positive moderate Spearman correlation was found with regard to the results on the phoneme level (*r* = 0.387, *p* < 0.01). Whereas all the other variables were correlated using Pearson’s correlation.

#### 4.3.2 Significant results on the word level

The results on the word level of listening comprehension correlate moderately with the cloze-tests results. A weaker correlation can be attested for the results of word level listening comprehension and the results of the vocabulary test. The amount of Russian the mothers talk to the participants and the amount of Russian the participants talk to the mothers also show a positive correlation with the results of the word level listening comprehension. Both the amount of Russian the mothers talk to the participants with a coefficient of *r* = 0.339 and the amount of Russian the participants use to communicate with their mothers, with a coefficient of *r* = 0.408, correlate moderately with the results on the word level of listening comprehension. Other significant correlations between listening comprehension of the word level and the amount of Russian the participants use in communication with their contact persons in general (*r* = 0.328), with siblings (*r* = 0.399) and their grandfathers (*r* = 0.323) could be attested. The results are shown in Table 10.

**Table 10:** Correlations of linguistic and background variables on the word level.

	Word level	Vocabulary	grammar	Mother to PT	PT to mother	PT to grandfather	PT to siblings
Vocabulary	0.383^**^						
Grammar	0.558^**^	0.673^**^					
Mother to PT	0.339^**^	0.508^**^	0.611^**^				
PT to mother	0.408^**^	0.428^**^	0.585^**^	0.683^**^			
PT to grandfather	0.323^**^	0.473^**^	0.646^**^	0.563^**^	0.561^**^		
PT to siblings	0.399^**^	0.404^**^	0.555^**^	0.455^**^	0.565^**^	0.322^*^	
PT to contact persons	0.328^**^	0.387^**^	0.505^**^	0.591^**^	0.783^**^	0.620^**^	0.691^**^

** < 0.01; * < 0.05.

In comparison to the phoneme level, the word level results depict a weak positive correlation with the amount of Russian used by the fathers for communication with the participants (*r* = 0.223, *p* < 0.05). Furthermore, weak correlations between the word level results and the participants’ use of Russian with the fathers (*r* = 0.296, *p* < 0.01) and grandmothers (*r* = 230, *p* < 0.05) could be attested. In terms of the education of the parents, the results show only a weak but significant correlation (*r* = 0.277, *p* = 0.038).

#### 4.3.3 Significant results on the sentence level

Very few significant correlations could be found with regard to the sentence level of listening comprehension. The analysis did not show any significant correlations concerning the participants’ communication language with any family members, including their mothers. This is a considerable difference to the phoneme, word, and text level.

A significant moderate correlation could only be attested between the sentence level of listening comprehension and the results of the grammar test with a coefficient of *r* = 0.340 (Table 11). Although the correlation with the vocabulary test results was significant, it was not strong enough with *r* = 0.240, *p* < 0.05. No significant correlation was attested between the results of the sentence level and the education of the parents.

**Table 11:** Correlations of linguistic and background variables on the sentence level.

	Sentence level
Grammar	0.340^**^

** < 0.01.

#### 4.3.4 Significant results on the text level

Regarding the text level of listening comprehension, a significant and strong positive correlation could be found with the results of the cloze-tests and a moderate correlation with the results of the vocabulary test. Again, the amount of Russian the mothers talk to the participants shows a moderate correlation with the results on this level of listening comprehension. Furthermore, correlations between the text comprehension results and the amount of Russian the participant talks to different contact persons can be attested. The amount of Russian the participants talk to their mothers and siblings shows a moderate correlation. The amount of Russian the participants use in communication with their contact persons in general and their grandfathers shows weak correlations with the results of text comprehension. A very weak correlation was detected between the participant’s speech with their fathers (*r* = 0.279, *p* < 0.01) and the amount of Russian input given by the fathers (*r* = 0.238, *p* < 0.05). With regard to the education of the parents the results show a positive moderate correlation by Spearman (*r* = 0.315, *p* < 0.05). All the other variables were correlated using Pearson and are displayed in Table 12.

**Table 12:** Correlations of linguistic and background variables on the sentence level.

	Text level	Vocabulary	Grammar	Mother to PT	PT to mother	PT to grandfather	PT to siblings	PT to contact persons
Vocabulary	0.472^**^							
Grammar	0.713^**^	0.673^**^						
Mother to PT	0.405^**^	0.508^**^	0.611^**^					
PT to mother	0.421^**^	0.428^**^	0.585^**^	0.683^**^				
PT to grandfather	0.318^**^	0.473^**^	0.646^**^	0.563^**^	0.561^**^			
PT to siblings	0.463^**^	0.404^**^	0.555^**^	0.455^**^	0.565^**^	0.322^*^		
PT to contact persons	0.338^**^	0.387^**^	0.505^**^	0.591^**^	0.783^**^	0.620^**^	0.691^**^	
Film and television in German	−0.333^**^	−0.202^*^	−0.357^**^	−0.346^**^	−0.281^**^	−0.240^*^	−0.384^**^	−0.360^**^

** < 0.01; * < 0.05.

In contrast to the phoneme, word, and sentence level, media consumption seems to play a more significant role at the text level of listening comprehension. Primarily, the consumption of films and television in Russian has a weak but significant relationship with the results of text level listening comprehension (*r* = 0.236, *p* < 0.05). On the other hand, the consumption of films and television in German has a moderate negative correlation with the results on this level of listening comprehension (*r* = −0.333, *p* < 0.01).

As for the consumption of social media and music, the analysis delivered no significant correlations. Nevertheless, a number of tendencies were discovered. Unsurprisingly, German media seem to correlate negatively with the results of Russian listening comprehension, whereas Russian media consumption seems to show rather positive correlations. However, the effects are very weak and not significant at all.

## 5 Discussion

The current study was devised to investigate listening comprehension and its influencing factors specifically in German-Russian simultaneous bilinguals aged 13–19 (*n* = 99) by considering the home- and majority language. The aim was to understand how language proficiency and input in Russian as a home language influence listening comprehension abilities. Additionally, the study aimed to explore potential differences among speakers in comprehension at various levels of the listening process and identify linguistic and background variables that may correlate with listening performance across different levels of listening. Although the listening comprehension in foreign- and second languages have been investigated in different language combinations, research on the entire process of listening by multilingual adolescents in specific language combinations and settings is still scarce (*cf.* Barac et al., 2016). With the present study it was tried to fill in this gap regarding the language combination of German as the majority and Russian as the home language in Germany.

With regard to the background variables and language proficiency, as anticipated, all the participants evaluated their knowledge of German higher than their knowledge of Russian (*cf.* Wald et al., 2023). Besides, the listening comprehension in Russian was the best estimated competence, which is in line with previous studies in this area of inquiry (e.g., Mehlhorn and Rutzen, 2020). In the German language, the participants estimated their receptive competences (listening and reading) higher than the productive ones. Whereas, in the Russian language both oral competences (listening and speaking) were stated to be higher, which is probably due to the knowledge of the Cyrillic alphabet or the lack thereof (*cf.* Schalley and Eisenchlas, 2020; Zabrodskaja et al., 2023). Furthermore, during the self-assessment of Russian skills, none of the participants evaluated oneself with the worst grade (6) in listening and speaking, which means that the participants must be able to understand and speak at least a little Russian, which can be confirmed by our tests.

The results of the vocabulary and grammar tests in Russian show that on average the participants coped better with the vocabulary than with the grammar (This may also be due to the test instrument). Moreover, the grammar test demonstrates a higher standard deviation, which goes in line with the results of the self-assessment test and with the outcomes in the previous studies on the competences in a home language (*cf.* Wald et al., 2023). The average high results in the grammar test could be partly explained by the fact that some of the participants have Russian classes at school.

In terms of language attitudes, Russian was given a high priority. This fact corresponds with the outcomes of the self-assessment tests and with the willingness to participate in the study at hand. Persons with a predominantly negative attitude toward the Russian language and a low assessment of their own knowledge would probably not take part in the study investigating competences in Russian voluntarily.

The results of the study show that most of the participants listen to Russian music and consume Russian-speaking media, TV, and films. However, the consumption of media in other languages, especially in English, was not the subject of the present study, which could probably have had an impact on the results as well. Furthermore, the participants appear to have several opportunities to receive input in Russian, e.g., from their parents, particularly from mothers, partly from their siblings, as well as out of the above-mentioned media. Thus, in their daily life, just over half of them speak more Russian than German within the family and 16% stated to speak no German at all (within the family). Regarding the language use in general, the participants use more Russian within the family, except their siblings, and more German outside the family (*cf.* Brehmer and Mehlhorn, 2015; Wald et al., 2023).

Concerning research question 1, the results show significant differences between the percentage of points on the phoneme level in comparison to word, sentence, and text level. However, no significant difference was found between word and sentence level. The highest percentage values were reached on the phoneme level while text comprehension had the lowest results in comparison.

Since the participants scored highest on the phoneme level, this could indicate that sound decoding is easier for them than word decoding, sentence parsing, or text comprehension as a total. This could be explained by the fact that phoneme level listening comprehension seems to be the least complex of all levels and sound decoding requires less working memory capacity (*cf.* Dietz, 2017) than processing mechanisms on higher levels. Besides, influencing factors like background knowledge, situational knowledge or even vocabulary knowledge play a less important role at this stage and more bottom-up processing is used (Field, 2008).

Another explanation for sound decoding being the easiest is that syllables and phonemes are learned earliest in life, as they are the smallest speech units (Bockmann et al., 2020). The results show that the participants receive a lot more input in Russian from their relatives and more German input outside the family. This indicates that in their first years of life when they are learning sounds and syllables the input in Russian is a lot greater than the German input, which means that the Russian language is dominant in this period (*cf.* Gervain and Werker, 2013; Byers-Heinlein et al., 2017). This could mean that Russian phonemes and syllables are learned more accurately at the beginning of childhood and lead to advanced skills in Russian sound decoding, while in other areas where more complex and systematic language is used, German already has a greater influence on the participants, and leads to less accurate word decoding and sentence parsing or even understanding of whole texts in Russian. The specific role of the first language and the time of exposure to the second language for sound decoding has already been underlined in previous studies and other language combinations (*cf.* Sebastián-Gallés et al., 2005).

No significant difference was found between the sentence and word level of comprehension which could mean that those two processes are similar in difficulty. This could be explained by the fact that rather short sentences were used to test sentence level listening comprehension and shorter sentences might have features in common with words, while the processing of longer sentences could be similarly difficult as text level processing.

Nevertheless, it must be mentioned that it was very difficult to find significant results for the sentence level of listening comprehension. So, a set of purely statistical factors such as sample size or sample selection could be responsible for the lack of significant results concerning the sentence level of processing (Field, 2013). Thus, it would make sense to conduct the study with an even bigger sample size.

The results on the text level were the lowest which indicates that the comprehension of the whole text is the most difficult in listening comprehension. This could be due to the amount of influencing factors like topic familiarity (Othman and Vanathas, 2017) or the complexity of the whole process, as a whole set of bottom-up and top-down processes has to be coordinated (Vandergrift, 2011; Marx and Roick, 2012).

Unfortunately, no influence of sentence comprehension on the results of text comprehension could be attested by the data. However, the linear regression model showed a significant influence of phoneme and word level processing on the text comprehension. This shows that both phoneme and word level influence the comprehension of the whole text, which shows a rather interactive character of the listening process (*cf.* Grosjean and Byers-Heinlein, 2018). The results of text comprehension might also depend on the topic since on the one hand topic familiarity has been identified as an important component for listening comprehension (Othman and Vanathas, 2017) and on the other hand language proficiency in a home language is domai-specific (Eisenchlas and Schalley, 2020).

Despite the numerous findings and possible justifications, it must be said that the comparability of a similar level of difficulty cannot be entirely guaranteed. Although attempts were made to use similarly difficult tasks at all levels, the effects seen by the comparison of the results might also show a difference in the level of difficulty of the tasks instead of the level of difficulty of the processes.

The analysis in 4.3 shows correlations with language proficiency on every level and therefore confirms findings from previous studies, such as Vandergrift and Baker (2015) and Marx and Roick (2012).

On phoneme level, both grammar and vocabulary test results showed significant strong correlations with the results of listening comprehension. However, on all other levels, the correlation of results was stronger with the grammar results than the vocabulary results.

Concerning the sentence level, only a few significant correlations were found. As already mentioned, it was a problem in general to get significant results in relation to the sound level of listening comprehension. Among others, this could be due to sample selection, sample size, or other statistical factors (Field, 2013).

Moderate correlations with the parents’ educational background were attested for the phoneme and text level, tendencies for correlations with the word level are also emerging but are too weak. These results show that the parents’ educational background is one considerable factor in connection with listening comprehension results. The findings are not surprising, since language proficiency in the home language is often influenced by the parents’ educational background (Schwartz, 2020).

Concerning the amount of Russian used by the participants no significant results could be attested with regard to contact persons outside of the family, e.g., friends, fellow students, and acquaintances (*cf.* Juvonen et al., 2020). Most correlations were detected between maternal input and the results of listening comprehension. The amount of Russian input the mothers give to the participants shows moderate correlations on phoneme, word, and sentence level. Moreover, the amount of Russian the participants use when communicating with their mothers, correlates with the results on word and sentence level. The role of maternal input has already been accounted for in several studies of home language research (Juvonen et al., 2020; Wald et al., 2023). In contrast, significant correlations with the input given by the fathers or the amount of Russian used by the participants in communication with their fathers were very weak or nonexistent.

Surprisingly, the language chosen by the participant while speaking to his grandfather seems to be connected to listening comprehension. Correlations with the amount of Russian use were spotted for phoneme level as well as for word and sentence level. This prominent role of language use by the grandfather cannot be explained that easily. The questions arises why correlations with the Russian use addressing the grandmother do not have the same relation to the listening comprehension proficiency. There are way weaker and not significant results in connection with language use by the participants with their grandmothers. However, as grandparents are often examined together and grandmother and grandfather are rarely separated when it comes to analyzing the impact of input in the home language (e.g., Riehl, 2018), these results may require further investigation.

Furthermore, tendencies for correlations with the amount of Russian used by the participants addressing their grandmothers and also fathers could be found in the data. These relations are weak and often not significant, but these tendencies could be reinforced by a bigger sample size.

A moderate correlation between the language chosen by the participants to communicate with their siblings and the listening comprehension results on word and sentence level was found. The participants stated to speak more German than Russian with their siblings, which is a common finding in different home language studies (*cf.* Barron-Hauwaert, 2011; Zabrodskaja et al., 2023). This is why it is particularly interesting that there is a link between listening comprehension skills and the amount of Russian spoken with these kinds of family members.

Another interesting finding was that most significant correlations were found on the text level. This is another confirmation of the complexity of this final product of the listening process (Field, 2008; Vandergrift, 2007).

The final product of listening comprehension was also the only level that showed any correlation with the use of media. Listening to music and the consumption of social media in both German and Russian had no correlation with the comprehension of the texts as a whole. In contrast, watching films and television in Russian had a positive correlation with the results of text level listening comprehension, although the correlation was very weak. An even stronger correlation was detected for the relationship between the consumption of German films and television and the text comprehension results in Russian. This correlation was moderate and negative. It remains questionable whether there is a causal connection between watching fewer films in German and understanding Russian text better. It is possible that this correlation reflects that reduced exposure to German media increases Russian input, which might benefit Russian listening comprehension.

Surprisingly, the attitudes toward the Russian language did not show any correlations with the scores on any level of listening comprehension, although the attitude toward the home language is often stated to be one of the factors for language proficiency in the home language (Mayer et al., 2020).

## 6 Limitations

The present study has a number of limitations that require acknowledgement. Firstly, it does not treat the psycholinguistic aspects of the listening comprehension research, as the focus of the present study is on sociolinguistics and the specific sociolinguistic context of the Russian language as a home language in Germany. Secondly, the use of the recorded speech in the study could be seen as a limitation. However, this procedure was developed in order to ensure equal conditions for all the participants and to archive a large number of the participants for better reliability. While the study focuses on the listening comprehension on different levels and possible corresponding linguistic and background variables, further research is needed to investigate the listening comprehension in Russian as a home language concerning natural speech, individual features of the speaker’s speech and one-time perception, which might also be important influence factors on the understanding of Russian speech by ear.

## Data Availability

The datasets presented in this article are not readily available because the data includes anonymized data. Requests to access the datasets should be directed to lgacs@uni-koblenz.de.

## Footnotes

^1^Data source MEZ - Multilingual Development: A Longitudinal Perspective; 2014–2019; Project coordination: Prof. Dr. Dr. h. c. mult. Ingrid Gogolin; © MEZ 2014, Mehrsprachigkeitsentwicklung im Zeitverlauf (MEZ) (2018, 2019a, 2019b, 2019c, 2019d).

^2^Data source MEZ - Multilingual Development: A Longitudinal Perspective; 2014–2019; Project coordination: Prof. Dr. Dr. h. c. mult. Ingrid Gogolin; © MEZ 2014.

^3^The German cloze tests originate from Baur and Spettmann (2008,2009).

## References

[ref1] Adamczak-Krysztofowicz S. Limbach C. (2019). “Hören” in Sprachdiagnostik Deutsch als Zweitsprache. eds. Jeuk S. Settinieri J. (Berlin: De Gruyter Mouton), 387–412.

[ref2] Ahlers T. Oberst T. Nentwig P. (2009). Redeanteile von Lehrern und Schülern im Chemieunterricht nach ChiK. Zeitschrift für Didaktik der Naturwissenschaften. 15, 331–342.

[ref3] Anstatt T. Mikić U. (2022). What does a receptive bilingual understand? Evidence from polish as a heritage language in Germany. Z. Slaw. 67, 355–388. doi: 10.1515/slaw-2022-0017

[ref4] Barac R. Moreno S. Bialystok E. (2016). Behavioral and electrophysiological differences in executive control between monolingual and bilingual children. Child Dev. 87, 1277–1290. doi: 10.1111/cdev.12538, PMID: 27133956 PMC4939124

[ref5] Barron-Hauwaert S. (2011). Bilingual Siblings. Language Use in Families. Bristol: Multilingual Matters.

[ref6] Baur R. S. Chlosta C. Roll H. (2019). “Zur Geschichte der Russlanddeutschen” in Handbuch des Russischen in Deutschland. eds. Witzlack-Makarevich K. Wulff N. (Berlin: Frank & Timme GmbH), 81–100.

[ref7] Baur R. Spettmann M. (2008). “Sprachstandsmessung und Sprachförderung mit dem C-Test” in Deutsch als Zweitsprache. eds. Ahrenholz B. Oomen-Welke I. (Baltmannsweiler: Schneider Verlag Hohengehren), 430–441.

[ref8] Baur R. Spettmann M. (2009). “Der C-Test als Instrument der Sprachdiagnose und Sprachförderung,” in Von der Sprachdiagnose zur Sprachförderung, eds. Lengyel D. Reich H. H. Roth H.-J. Döll M. (Münster: Waxmann), 115–127.

[ref9] Becker-Mrotzek M. Vogt R. (2009). Unterrichtskommunikation. Unterrichtskommunikation: Linguistische Analysemethoden und Forschungsergebnisse. Berlin: Walter de Gruyter.

[ref10] Belgrad J. Eriksson B. Pabst-Weinschenk M. Vogt R. (2008). Die Evaluation von Mündlichkeit. Kompetenzen in den Bereichen Sprechen, Zuhören und Szenisch Spielen. Didaktik Deutsch. 13, 20–45.

[ref11] Bialystok E. Craik F. I. (2022). How does bilingualism modify cognitive function? Attention to the mechanism. Psychon. Bull. Rev. 29, 1246–1269. doi: 10.3758/s13423-022-02057-5, PMID: 35091993

[ref12] Bockmann A. K. Sachse S. Buschmann A. (2020). “Sprachentwicklung im Überblick” in Sprachentwicklung: Entwicklung-Diagnostik-Förderung im Kleinkind- und Vorschulalter (Berlin: Springer), 3–44.

[ref13] Brehmer B. Mehlhorn G. (2015). Russisch als Herkunftssprache in Deutschland. Ein holistischer Ansatz zur Erforschung des Potenzials von Herkunftssprachen. Zeitschrift Fremdsprachenforschung. 26, 85–123.

[ref14] Brüggemann M. (2019). “Sootečestvenniki, Diaspora und Russkij mir: Die externe Sprachpolitik Russlands nach 1991” in Handbuch des Russischen in Deutschland. Migration-Mehrsprachigkeit-Spracherwerb. eds. Witzlack-Makarevich K. Wulff N. (Berlin: Frank & Timme GmbH), 199–220.

[ref15] Byers-Heinlein K. (2018). “Speech perception” in The listening bilingual: Speech perception, comprehension, and bilingualism. ed. Grosjean F. (Oxford: John Wiley & Sons), 153–175.

[ref16] Byers-Heinlein K. Morin-Lessard E. Lew-Williams C. (2017). Bilingual infants control their languages as they listen. Proc. Natl. Acad. Sci. 114, 9032–9037. doi: 10.1073/pnas.1703220114, PMID: 28784802 PMC5576790

[ref17] Chen A. (2010). Effects of listening strategy training for EFL adult listeners. J. Asia TEFL. 7, 135–169.

[ref18] Cohen J. (1988). Statistical power analysis for the behavioral sciences. 2nd Edn. New York, NY: Routledge.

[ref19] Connaughton-Crean L. Ó’Duibhir P. (2017). Home language maintenance and development among first generation migrant children in an Irish primary school: an investigation of attitudes. J. Home Lang. Res. 2, 22–39. doi: 10.16993/jhlr.29

[ref20] Cooper R. L. Fowles B. R. Givner A. (1969). Listening comprehension in a bilingual community. Mod. Lang. J. 53, 235–241. doi: 10.1111/j.1540-4781.1969.tb07026.x

[ref21] Cutler A. (2012). Native listening. Language experience and the recognition of spoken words. Cambridge: The MIT Press.

[ref22] Cutler A. Broersma M. (2005). “Phonetic precision in listening” in A figure of speech. A festschrift for John laver. eds. Hardcastle W. J. Beck M. (London: Lawrence Erlbaum Associates), 63–91.

[ref23] Diekmann A. (2017). Empirische Sozialforschung: Grundlagen, Methoden, Anwendungen. Reinbek: Rowohlt Verlag GmbH.

[ref24] Dietz G. (2017). “Mentale Prozesse beim mutter- und fremdsprachlichen Hören und Konsequenzen für die Hörverstehensdidaktik,” in DaZu und DaFür. Neue Perspektiven für das Fach Deutsch als Zweit- und Fremdsprache zwischen Flüchtlingsintegration und weltweitem Bedarf, eds. Venanzio L. Di Lammers I. Roll H.. (Göttingen: Universitätsverlag Göttingen), 97–116.

[ref25] Dietz B. Roll H. (2019). “Die Einwanderung aus der Sowjetunion und ihren Nachfolgestaaten” in Handbuch des Russischen in Deutschland. Migration– Mehrsprachigkeit – Spracherwerb. eds. Witzlack-Makarevich K. Wulff N. (Berlin: Frank & Timme GmbH), 101–114.

[ref26] Eberly L. E. (2007). “Multiple linear regression,” in topics in biostatistics. Methods Mol. Biol. 404, 165–187. doi: 10.1007/978-1-59745-530-5_9 18450050

[ref27] Eckhardt A. G. (2020). “Hörverstehen in der Zweitsprache Deutsch” in Deutsch als Zweitsprache. eds. Ahrenholz B. Oomen-Welke I. (Hohengehren: Schneider Verlag), 327–340.

[ref28] Edele A. (2016). Die Rolle herkunftssprachlicher Kompetenz und kultureller Identität für den Bildungserfolg von Heranwachsenden aus zugewanderten Familien. Erziehungswissenschaft und Psychologie. [Doctoral dissertation]. Berlin: Freie Universität Berlin.

[ref29] Edele A. Schotte K. Hecht M. Stanat P. (2012). Listening comprehension tests of immigrant students’ first language (L1) Russian and Turkish in grade 9: Scaling procedure and results (NEPS working paper No. 13). Bamberg: Otto-Friedrich-Universität, National Educational Panel Study.

[ref30] Edele A. Schotte K. Stanat P. (2015). Listening comprehension tests of immigrant students’ first languages (L1) Russian and Turkish in grade 9: Extended report of test construction and validation (NEPS working paper No. 57). Bamberg: Leibnitz Institute for Educational Trajectories, National Educational Panel Study.

[ref31] Eisenchlas S. A. Schalley A. C. (2020). “Making sense of “home language” and related concepts” in Handbook of home language maintenance and development: social and affective factors. eds. Schalley A. C. Eisenchlas S. A. (Berlin: De Gruyter Mouton), 17–37. doi: 10.1515/9781501510175

[ref32] Field J. (2008). Listening in the language classroom. ELT J. 64, 331–333. doi: 10.1093/elt/ccq026

[ref33] Field A. (2013). Discovering statistics using IBM SPSS statistics. London: SAGE Publications.

[ref34] Gervain J. Werker J. F. (2013). Prosody cues word order in 7-month-old bilingual infants. Nat. Commun. 4, 1490–1496. doi: 10.1038/ncomms2430, PMID: 23411502

[ref35] Gogolin I. Klinger T. Lagemann M. Schnoor B. (2017). Indikation, Konzeption und Untersuchungsdesign des Projekts Mehrsprachigkeitsentwicklung im Zeitverlauf (MEZ). Hamburg: Universität Hamburg.

[ref36] Grenoble L. A. (2003). Language policy in the Soviet Union. Dordrecht: Kluwer Academic Publishers.

[ref37] Grosjean F. (2018). “Spoken word recognition” in The listening bilingual: speech perception, comprehension, and bilingualism. ed. Byers-Heinlein K. (Oxford: John Wiley & Sons), 65–85.

[ref38] Grosjean F. Byers-Heinlein K. (2018). “Speech perception and comprehension” in The listening bilingual: Speech perception, comprehension, and bilingualism (Oxford: John Wiley & Sons), 25–42.

[ref39] Hecker K. Südkamp A. Leser C. Weinert S. (2015). Entwicklung eines Tests zur Erfassung von Hörverstehen auf Textebene bei Schülerinnen und Schülern der Klassenstufe 9 (NEPS Working Paper No. 53). Bamberg: Leibnitz-Institut für Bildungsverläufe, Nationales Bildungspanel.

[ref40] Imhof M. (2010). “Zuhören Lernen und Lehren. Psychologische Grundlagen zur Beschreibung und Förderung von Zuhörkompetenzen in Schule und Unterricht” in Zuhörkompetenz in Unterricht und SchuleBeiträge aus Wissenschaft und Praxis. ed. Bernius V. (Göttingen: Vandenhoeck & Ruprecht), 15–30.

[ref41] Islam M. R. (2018). Sample size and its role in central limit theorem (CLT). IJPM 4, 1–7. doi: 10.31295/ijpm.v1n1.42

[ref42] Jiang H. Farquharson K. (2018). Are working memory and behavioral attention equally important for both reading and listening comprehension? A developmental comparison. Read. Writ. 31, 1449–1477. doi: 10.1007/s11145-018-9840-y, PMID: 30147241 PMC6096896

[ref43] Juvonen P. Eisenchlas S. A. Roberts T. Schalley A. C. (2020). “Researching social and affective factors in home language maintenance and development: a methodology overview” in Handbook of home language maintenance and development: social and affective factors. eds. Schalley A. C. Eisenchlas S. A. (Berlin: De Gruyter), 38–62.

[ref44] Karpava S. (2022). The interrelationship of family language policies, emotions, socialisation practices and language management strategies. J. Home Lang. Res. 5, 1–23. doi: 10.16993/jhlr.44

[ref46] Lanza E. (2021). The family as a space: multilingual repertoires, language practices and lived experiences. J. Multiling. Multicult. Dev. 42, 763–771. doi: 10.1080/01434632.2021.1979015

[ref47] Lengyel D. (2012). “Unterrichtsinteraktion in sprachlich heterogenen Klassen” in Interkulturelle Pädagogik und Sprachliche Bildung. ed. Fürstenau S. (Wiesbaden: Springer), 143–161.

[ref48] Marx A. (2016). Hören und Verstehen: Struktur und Determination des Hörverstehens bei Schülerinnen und Schülern mit Deutsch als Erst- bzw. Zweitsprache. [Doctoral dissertation]. Berlin: Freie Universität Berlin.

[ref49] Marx A. Roick T. (2012). Prädiktoren des Hörverstehens bei Jugendlichen deutscher und Jugendlichen nichtdeutscher Herkunftssprache 3Dieser Beitrag wurde unter der geschäftsführenden Herausgeberschaft von Jens Möller angenommen. Zeitschrift für Pädagogische Psychologie. 26, 121–134. doi: 10.1024/1010-0652/a000067

[ref50] Mayer E. Sánchez L. Camacho J. Alzza C. R. (2020). “The drivers of home language maintenance and development in indigenous communities” in Handbook of home language maintenance and development: social and affective factors. eds. Schalley A. C. Eisenchlas S. A. (Berlin: De Gruyter), 312–336.

[ref51] Mehlhorn G. Rutzen K. M. (2020). “Didaktische Prinzipien für den Unterricht mit Herkunftssprachen-und Fremdsprachenlernenden” in Handbuch Mehrsprachigkeit und Bildung. eds. Gogolin I. Hansen A. McMonagle S. Rauch D. (Wiesbaden: Springer), 219–225.

[ref9001] Mehrsprachigkeitsentwicklung im Zeitverlauf (MEZ). (2018): Variablenhandbuch Querschnittsdaten: Elternfragebogen. Hamburg (Universität Hamburg, MEZ-Projekt), Mimeo.

[ref9002] Mehrsprachigkeitsentwicklung im Zeitverlauf (MEZ). (2019a): Variablenhandbuch 1 (1. Erhebungswelle). Hamburg (Universität Hamburg, MEZ-Projekt), Mimeo.

[ref9003] Mehrsprachigkeitsentwicklung im Zeitverlauf (MEZ). (2019b): Variablenhandbuch 2 (2. Erhebungswelle). Hamburg (Universität Hamburg, MEZ-Projekt), Mimeo.

[ref9004] Mehrsprachigkeitsentwicklung im Zeitverlauf (MEZ). (2019c): Variablenhandbuch 3 (3. Erhebungswelle). Hamburg (Universität Hamburg, MEZ-Projekt), Mimeo.

[ref9005] Mehrsprachigkeitsentwicklung im Zeitverlauf (MEZ). (2019d): Variablenhandbuch 4 (4. Erhebungswelle). Hamburg (Universität Hamburg, MEZ-Projekt), Mimeo.

[ref52] Melo-Pfeifer S. (2015). The role of the family in heritage language use and learning. Impact on heritage language policies. Int. J. Biling. Educ. Biling. 18, 26–44. doi: 10.1080/13670050.2013.868400

[ref53] Namaziandost E. Neisi L. Mahdavirad F. Nasri M. (2019). The relationship between listening comprehension problems and strategy usage among advance EFL learners. Psychology 6:1338. doi: 10.1080/23311908.2019.1691338

[ref54] Nix J. M. L. (2016). Measuring latent listening strategies. Development and validation of the EFL listening strategy inventory. System 57, 79–97. doi: 10.1016/j.system.2016.02.001

[ref55] Othman J. Vanathas C. (2017). Topic familiarity and its influence on listening comprehension. English Teacher 34, 19–32.

[ref56] Park E. Cho M. Ki C. S. (2009). Correct use of repeated measures analysis of variance. Korean J. Lab. Med. 29, 1–9. doi: 10.3343/kjlm.2009.29.1.1 19262072

[ref57] Riehl C. M. (2015). Claudia Maria Riehl. 2014. Mehrsprachigkeit. Eine Einführung. Zeitschrift für Rezensionen zur germanistischen Sprachwissenschaft. 7, 173–176. doi: 10.1515/zrs-2015-0033

[ref58] Riehl C. M. (2018). “Mehrsprachigkeit in der Familie und im Lebensalltag” in Deutsch als Zweitsprache: Migration - Spracherwerb – Unterricht. eds. Haar A. K. Liedke M. (Stuttgart: Metzler), 27–60.

[ref59] Ritter A. (2021). Language choice and language contact in print advertisements for Russian-speaking immigrants in Germany. Russian J. Ling. 25, 958–980. doi: 10.22363/2687-0088-2021-25-4-958-980

[ref60] Schalley A. C. Eisenchlas S. A. (2020). Handbook of home language maintenance and development: Social and affective factors, Vol. 18. Berlin: Walter de Gruyter.

[ref61] Schwartz M. (2020). “10 strategies and practices of home language maintenance” in Handbook of home language maintenance and development. eds. Schalley A. C. Eisenchlach S. A. (Berlin: De Gruyter Mouton), 194–217.

[ref62] Schwartz M. Verschik A. (2013). “Achieving success in family language policy: parents, children and educators in interaction” in Successful family language policy: Parents, children and educators in interaction, Vol. 7 (Dordrecht: Springer Netherlands), 1–20.

[ref63] Sebastián-Gallés N. Echeverría S. Bosch L. (2005). The influence of initial exposure on lexical representation: comparing early and simultaneous bilinguals. J. Mem. Lang. 52, 240–255. doi: 10.1016/j.jml.2004.11.001

[ref64] Stanat P. Schipolowski S. Schneider R. Sachse K. A. Weirich S. Henschel S. (2022). IQB-Bildungstrend 2021. Kompetenzen in den Fächern Deutsch und Mathematik am Ende der 4. Jahrgangsstufe im dritten Ländervergleich. Münster: Waxmann Verlag.

[ref65] Suemith M. E. (2011). The communicative language teaching approach: theory and practice. Magister Sci. 30, 1–9. doi: 10.33508/mgs.v0i30.627

[ref66] Valentini A. Serratrice L. (2023). Longitudinal predictors of listening comprehension in bilingual primary school-aged children. Lang. Learn. 73, 5–46. doi: 10.1111/lang.12513

[ref67] Van Dijk C. Van Wonderen E. Koutamanis E. Kootstra G. J. Dijkstra T. Unsworth S. (2022). Cross-linguistic influence in simultaneous and early sequential bilingual children: a Meta-analysis. J. Child Lang. 49, 897–929. doi: 10.1017/S0305000921000337, PMID: 34183085

[ref68] Vandergrift L. (2006). Second language listening: listening ability or language proficiency? Mod. Lang. J. 90, 6–18. doi: 10.1111/j.1540-4781.2006.00381.x

[ref69] Vandergrift L. (2007). Recent developments in second and foreign language listening comprehension research. Lang. Teach. 40, 191–210. doi: 10.1017/S0261444807004338

[ref70] Vandergrift L. (2011). “Second language listening: presage, process, product, and pedagogy” in Handbook of research in second language teaching and learning. ed. Hinkel E. (New York: Routledge), 455–471.

[ref71] Vandergrift L. Baker S. (2015). Learner variables in second language listening comprehension: an exploratory path analysis. Lang. Learn. 65, 390–416. doi: 10.1111/lang.12105

[ref72] Wald V. Ritter A. Kurbangulova T. (2023). “Zweisprachigkeit und Sprachmanagement in russischsprachigen Familien in Deutschland” in Linguistische Beiträge zur Slavistik. eds. Clasmeier C. Golbek J. (Lausanne: Peter Lang), 177–191.

[ref73] Wilson I. Kaneko E. Lyddon P. Okamoto K. Ginsburg J. (2011). Nonsense-syllable sound discrimination ability correlates with second language (L2) proficiency. Listening 495, 50–58.

[ref45] Zabrodskaja A. Meir N. Ritter A. Ringblom N. Karpava S. (2024). “Access to heritage and majority language education during the COVID-19 pandemic: new experiences and opportunities” in Family and School Involvement in Multilingual Education and Heritage Language Development. ed. Karpava S. (Leiden: Brill), 240–261.

[ref74] Zabrodskaja A. Meir N. Karpava S. Ringblom N. Ritter A. (2023). Family language policies of multilingual families during the COVID-19 pandemic: evidence from Cyprus, Estonia, Germany, Israel, and Sweden. Languages 8:263. doi: 10.3390/languages8040263

